# Complete Genome Sequence of *Bacillus toyonensis* BCT-7112^T^, the Active Ingredient of the Feed Additive Preparation Toyocerin

**DOI:** 10.1128/genomeA.01080-13

**Published:** 2013-12-19

**Authors:** Guillermo Jiménez, Anicet R. Blanch, Javier Tamames, Ramon Rosselló-Mora

**Affiliations:** Rubinum, S.A., Rubí, Spaina; Department of Microbiology, University of Barcelona, Barcelona, Catalonia, Spainb; Systems Biology Department, Centro Nacional de Biotecnología, Madrid, Spainc; Marine Microbiology Group, Department of Ecology and Marine Resources, Mediterranean Institute for Advanced Studies (IMEDEA) (CSIC-UIB), Esporles, Illes Balears, Spaind

## Abstract

Strain BCT-7112, previously identified as *Bacillus cereus* var. *toyoi*, is the type strain of the species *Bacillus toyonensis*, a novel species of the *B. cereus* group. The complete genome of this strain, which is the active ingredient of the feed additive preparation Toyocerin, has been sequenced and annotated to reveal the genetic properties of this probiotic organism with a long history of safe use in animal nutrition.

## GENOME ANNOUNCEMENT

Strain BCT-7112 is the type strain of the newly classified species *Bacillus toyonensis* (1). This species is a member of the *B. cereus* group (2), which currently also comprises the species *B. cereus, B. thuringiensis, B. anthracis, B. mycoides, B. pseudomycoides, B. weihenstephanensis*, and *B. cytotoxicus*. All the members of the group are considered soilborne organisms, and some species of this group are considered pathogens (3), whereas others are considered relevant as either biological insecticides (2) or probiotic microorganisms in animal feed (4). The strain BCT-7112^T^ was isolated in Japan in 1966, and it has been used as a probiotic in animal feed since 1975 (1).

The draft genome sequencing of strain BCT-7112^T^ was performed at Lifesequencing SL (Valencia, Spain) using a shotgun and paired-end strategy with 454 technology, and the genome sequence was closed by LGC Genomics GmbH (Berlin, Germany). *De novo* assembly was performed using Newbler assembler version 2.6. Gaps within and between the scaffolds were closed by primer walking, PCR amplification, and standard Sanger sequencing using BigDye 3.1 Terminator chemistry on an ABI3739XL genetic analyzer. Genome sequence alignments were done using progressiveMauve (5).

The complete genome of BCT-7112^T^ is 5.03 Mb in size, containing one single circular chromosome (4,940,474 bp) and two circular plasmids, pBCT77 (76,974 bp) and pBCT8 (7,971 bp). The G+C content of the chromosome is 35.6%, and the G+C contents of the plasmids are 32.9% and 31.4%, respectively. The chromosome contained 5,232 coding DNA sequences (CDS), 9 rRNA operons, and 106 tRNA sequences.

Two antibiotic resistance genes are present in the genome of BCT-7112^T^, *catQ* and *tetM*, encoding resistances to chloramphenicol and tetracycline, respectively. However, orthologues of both genes appear in all genome sequences available for the putative members of the species (1), indicating a common ancestry. Both genes are located in a structurally conserved region of the chromosome, and no genetic mobile elements or other horizontal gene transfer mechanisms have been detected in their respective flanking regions. Furthermore, other features, such as G+C content (*catQ* gene G+C content, 29.8%; *tetM* gene G+C content, 37.4%) and the codon adaptation index (CAI) (*catQ* gene CAI, 0.76; *tetM* gene CAI, 0.69) (6), confirm the intrinsic nature of such genes.

The genome of *B. toyonensis* BCT-7112^T^ shows relevant differences from the *B. cereus* food-poisoning strains regarding the presence of genes considered to be the causative agents of gastrointestinal diseases (7). The Nhe (nonhemolytic enterotoxin) and Hbl (hemolysin BL) operons present in the genome of *B. toyonensis* may be nonfunctional, since some of their toxin components show important amino acid changes at the level of the Sec type signal peptide (8). In addition, the operon encoding cereulide synthase lacks the genes *cesD, cesH, cesP*, and *cesT*, and therefore, it is not expected to be functional. Finally, the *cytK* gene is absent in the genome of *B. toyonensis*. These findings at the genomic level are in accordance with the proven safety of the use of this bacterium as a probiotic in studies carried out *in vitro* and *in vivo* with laboratory animals, livestock animals, and humans (4, 9).

### Nucleotide sequence accession numbers.

The completed genome sequence of *B. toyonensis* BCT-7112^T^ has been deposited in the DDBJ/EMBL/GenBank database. The accession number of the chromosome is CP006863, and the accession numbers for plasmids pBCT77 and pBCT8 are CP006864 and CP006865, respectively.
